# Artificial Intelligence in Aesthetic Medicine: Applications, Challenges, and Future Directions

**DOI:** 10.1111/jocd.70241

**Published:** 2025-06-12

**Authors:** Mohammed Saleh Al‐Dhubaibi, Ghada Farouk Mohammed, Lina Mohammed Atef, Saleh Salem Bahaj, Ahmed Mohammed Al‐Dhubaibi, Ahmad Mohammed Bukhari

**Affiliations:** ^1^ Department of Dermatology, College of Medicine Shaqra University Dawadmi Saudi Arabia; ^2^ Department of Dermatology, Venereology, and Sexology, Faculty of Medicine Suez Canal University Ismailia Egypt; ^3^ Department of Microbiology and Immunology Faculty of Medicine and Health Sciences, Sana'a University Yemen; ^4^ Medical Student, College of Medicine Marmara University Istanbul Turkey; ^5^ Medical Student, College of Medicine Umm Al‐Qua University Makkah Saudi Arabia

**Keywords:** aesthetic medicine, artificial intelligence, machine learning

## Abstract

**Background:**

Artificial Intelligence (AI) is transforming healthcare by enhancing diagnostics, treatment personalization, and operational efficiency. In aesthetic medicine—a field blending medical expertise with artistic judgment—AI is increasingly being used to improve precision, optimize treatment outcomes, and personalize patient care. However, its integration presents both opportunities and ethical challenges, necessitating a critical evaluation of its role in this evolving field.

**Objective:**

This study examines AI's applications in aesthetic medicine, focusing on its role in facial analysis, robotic‐assisted procedures, predictive patient outcome modeling, and personalized treatment planning. Additionally, it explores ethical concerns, algorithmic biases, data privacy issues, and regulatory challenges affecting AI adoption in aesthetic practices.

**Methods:**

A comprehensive review of AI‐driven technologies in aesthetic medicine was conducted, analyzing literature on machine learning (ML), deep learning, and computer vision applications. Case studies on AI‐assisted facial symmetry analysis, robotic hair transplantation, and predictive analytics in patient care were examined to evaluate AI's effectiveness and limitations.

**Results:**

AI enhances aesthetic procedures by improving diagnostic accuracy, offering virtual simulations of treatment outcomes, and enabling hyper‐personalized treatment plans based on patient data. AI‐driven chatbots and virtual assistants streamline patient interactions, while robotic systems assist in precision‐based tasks such as laser treatments and hair restoration. However, challenges such as biased training data, lack of transparency in AI decision‐making, and inconsistencies in regulatory approvals hinder widespread adoption.

The integration of AI in aesthetic medicine presents a paradigm shift from traditional approaches to data‐driven, personalized interventions. However, ethical concerns such as data privacy, informed consent, and algorithmic fairness must be addressed. Overreliance on AI may diminish the human‐centric approach essential in aesthetic procedures, where patient expectations and subjective perceptions of beauty play a crucial role. Collaboration between technologists, clinicians, and policymakers is necessary to develop standardized AI guidelines that ensure fairness, safety, and efficacy.

**Conclusion:**

AI has the potential to revolutionize aesthetic medicine by improving precision, efficiency, and patient satisfaction. However, its successful implementation requires balancing technological advancements with ethical considerations and regulatory frameworks. Future research should focus on integrating AI with emerging technologies such as augmented reality (AR) and genomic‐based personalization to enhance aesthetic outcomes while maintaining transparency and patient trust.

## 1 Introduction

Artificial intelligence (AI) is revolutionizing health care at breakneck pace with capabilities never previously seen in diagnosis, treatment suited to the unique individual, and back‐end productivity [1]. From machine‐learning software that identifies tumors on X‐rays to natural language processing software that enables automated clinical documentation, AI is augmenting human knowledge, reducing errors and costs. Its capacity to process enormous data sets—from genomic profiles to real‐time monitoring of patients—makes predictive analysis and precision medicine possible, essentially transforming the way care is given [2]. With increased adoption, its impact is also spreading beyond the realm of conventional medicine to specialist areas such as dermatology, plastic surgery, and cosmetic treatment, where its potential for innovation is immense but complex [3].

Aesthetic medicine, a medical specialty focused on enhancing physical appearance through minimally invasive or nonsurgical methods, is all about symmetry, proportion, and patient satisfaction [4]. These include procedures such as Botox, dermal fillers, laser work, and body contouring intended to address aging, skin wellness, or congenital asymmetry, often marrying medical expertise with artistic sensibility [5]. In contrast to general medicine, aesthetic medicine takes place at the interface of science and personal ideals of beauty, so practitioners need to reconcile technical competence with an appreciation of individual patients' wishes [6]. This specific context makes the domain highly susceptible to AI incorporation because technology can leverage objectivity, customization, and results while negotiating intricate aesthetic values [7].

AI is revolutionizing aesthetic medicine by facilitating data‐driven treatment planning, predictive analytics, and hyper‐personalized patient experiences—but its adoption presents significant ethical and technical challenges [8]. Algorithms now power virtual simulations of postprocedure outcomes, recommend personalized skincare routines, and assist robotic instruments in performing precise interventions [3]. But issues such as biased training data, patient privacy, and overautomation need careful scrutiny [9]. As the field evolves, stakeholders must harmonize AI's transformative potential with safeguards to ensure equitable, transparent, and human‐centered care in this deeply personal domain [10].

### 1.1 Research Objectives

This study aims to examine the current applications of AI in aesthetic medicine, including facial simulation, robotic‐assisted procedures, and predictive analytics. Identify key challenges—spanning ethical concerns, regulatory gaps, and technological limitations—that hinder responsible AI deployment. Propose actionable strategies to balance innovation with human‐centric care, emphasizing transparency, inclusivity, and interdisciplinary collaboration.

### 1.2 Research Problem

The rapid integration of AI into aesthetic medicine has outpaced critical evaluations of its ethical, technical, and societal implications. While AI offers transformative tools for facial analysis, personalized treatments, and outcome prediction, challenges such as algorithmic bias, data privacy risks, and overreliance on automation threaten patient safety and equity. The field lacks standardized guidelines to govern AI adoption, creating disparities in care quality and eroding trust in technology‐driven interventions.

### 1.3 Significance of the Study

The significance of this study lies in its capacity to guide the responsible development of AI technologies in an area where subjective standards of beauty and objective medical practice converge. By bridging critical gaps in regulation, fairness, and clinical practice, the research aims to create guidelines that promote patient well‐being while harnessing the transformative power of AI. Findings will be most helpful to active practitioners seeking to incorporate AI utilities into practice, developers designing aesthetic tools, and lawmakers who formulate suitable regulatory frameworks. Ultimately, the current investigation enhances the discourse at the population level around medical AI by bringing clarity on certain distinct concerns relevant to aesthetic medicine, where innovation facilitated through technology is weighed precariously with considerations around artistic appreciation as well as patient welfare.

## 2 Methodology

This narrative review synthesizes qualitative insights from peer‐reviewed literature, industry reports, and clinical case studies (2015–2024) to explore AI's role in aesthetic medicine. Using thematic analysis, we evaluate key applications (e.g., facial simulation, robotic surgery), ethical challenges (bias, privacy), and emerging trends (AR/VR integration, genomics). Sources were selected from PubMed, IEEE Xplore, and dermatology journals, prioritizing high‐impact studies and paradigm cases that illustrate AI's transformative potential and limitations. The analysis emphasizes critical discourse around human–AI collaboration, regulatory gaps, and patient‐centered outcomes, offering a comprehensive yet interpretive assessment of the field's evolution.

### 2.1 Understanding AI in Aesthetic Medicine

AI is computer applications designed to accomplish tasks that generally require human intellect, such as decision‐making, pattern recognition, and image interpretation [11]. Machine learning in AI enables algorithms to improve over time with experience without programming, and computer vision enables computers to interpret and analyze visual data, such as images or video [12]. Deep learning, a subset of ML, uses multi‐layered neural networks to process complex data—like facial features or skin textures—with remarkable accuracy [13]. Together, these technologies form the backbone of AI's capability to mimic and augment human expertise, particularly in fields requiring precision and adaptability [14].

In aesthetic medicine, AI is relevant because it can improve precision, personalization, and automation [15]. Facial symmetry and skin conditions are examined by computer vision algorithms with pixel‐perfect precision, allowing clinicians to craft treatments specific to individual anatomies [16]. Machine learning models examine patient histories, genetic information, and lifestyle variables to forecast best results for procedures such as filler injections or laser treatments, providing hyperpersonalized care [17]. And as automation streamlines workflows—from AI chatbots booking appointments to robotic equipment assisting in minimum invasions—the practitioner is then free to fret about innovative and complex tasks. This triple threat of precision, customization, and automation makes AI the game‐changer force in a field where art and medical science converge [18].

**FIGURE 1:**
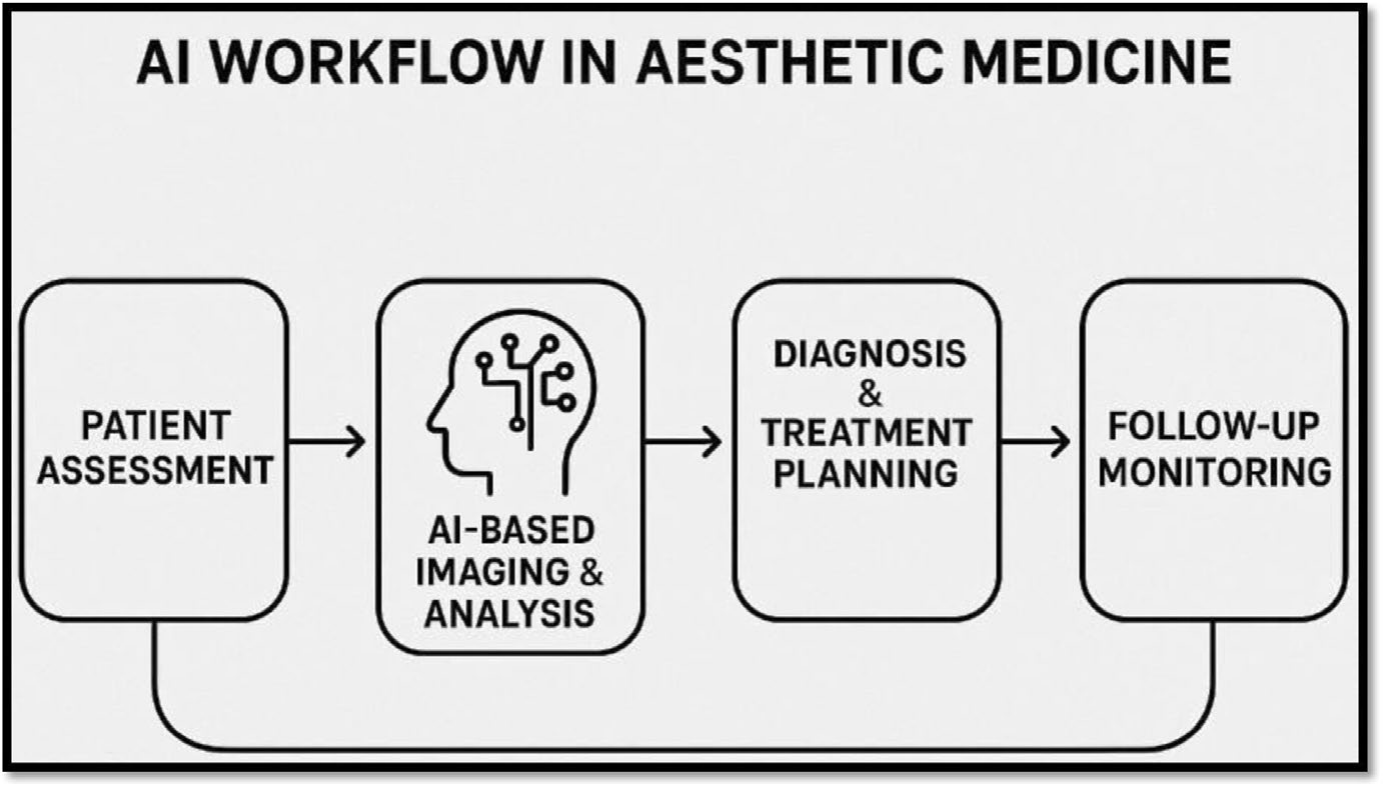
AI workflow in aesthetic medicine.

Adoption trends in the present day are looking at increased adoption of AI in every kind of aesthetic treatment. Technology like 3D facial mapping software, virtual “before‐and‐after” planners, and AI‐powered skin diagnostic apps (like Haut.AI or Perfect Corp) are now the norm at clinics, streamlining patient consultations and treatment planning [19]. Startups and established technology firms such as Canfield Scientific and ModiFace, which is owned by L'Oréal, are working with physicians to develop platforms that read aging patterns or forecast surgical outcomes [20]. Adoption varies globally, with technologically advanced regions such as South Korea and the United States leading AI‐assisted robotic surgeries and tele‐aesthetics, while regulatory hurdles in the European Union and elsewhere restrain adoption. Despite challenges, the trend is toward AI as an indispensable tool, reshaping patient and clinical expectations in aesthetic medicine (Figure 1) [21].

## 3 Application of AI in Aesthetic Medicine

### 3.1 Facial Analysis and Simulation

**FIGURE 2:**
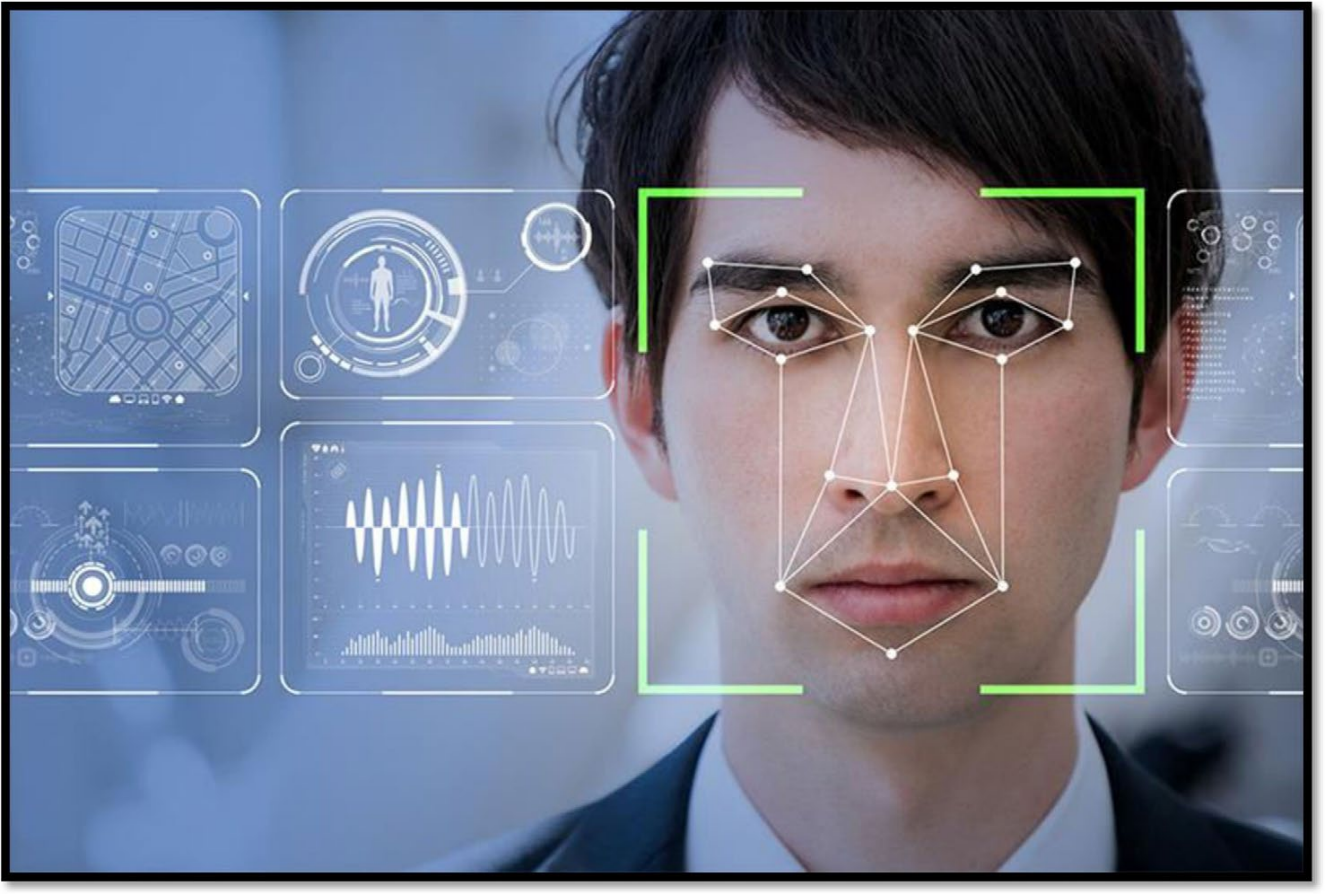
AI facial analysis [26].

AI has revolutionized facial analysis and simulation in aesthetic medicine by offering clinicians objective tools to evaluate symmetry, age pattern prediction, and 3D modeling of patient anatomy [22]. Facial landmarks are analyzed with sophisticated computer vision algorithms to identify asymmetry or disproportions, enabling clinicians to design treatments to restore balance—for example, dermal fillers or botulinum toxin injections [23, 24]. Deep learning algorithms predict how a patient's characteristics may age in the future and guide preventative interventions. Additionally, 3D modeling and AR allow patients to visualize possible outcomes of procedures like rhinoplasty or cheek enhancement in interactive “before‐and‐after” virtual reality simulations [25]. These technologies are used by platforms like ModiFace or Crisalix to optimize patient–clinician communication, create realistic expectations, and optimize satisfaction by bridging the gap between artistic intention and achievable results (Figure 2) [27].

### 3.2 Personalized Treatment Planning

Computer‐optimized personalized treatment planning is a break from the past one‐size‐fits‐all approach to hyperindividualized treatment. Machine learning algorithms analyze diverse data points—skin type, genetic predisposition, lifestyle factors, and past treatment histories—to recommend optimal treatments, either laser treatments for pigment or fillers to round out [28]. For instance, AI‐driven software like Haut.AI analyzes skin texture and moisture levels to deliver customized skincare regimens, while tools like Allergan's SkinMedica utilize predictive analytics to match patients with appropriate chemical peels or serums. Data‐driven precision minimizes trial‐and‐error, decreases side effects, and keeps interventions aligned with personal aesthetic needs and biological factors [29].

### 3.3 Robotic‐Assisted Procedures

**FIGURE 3:**
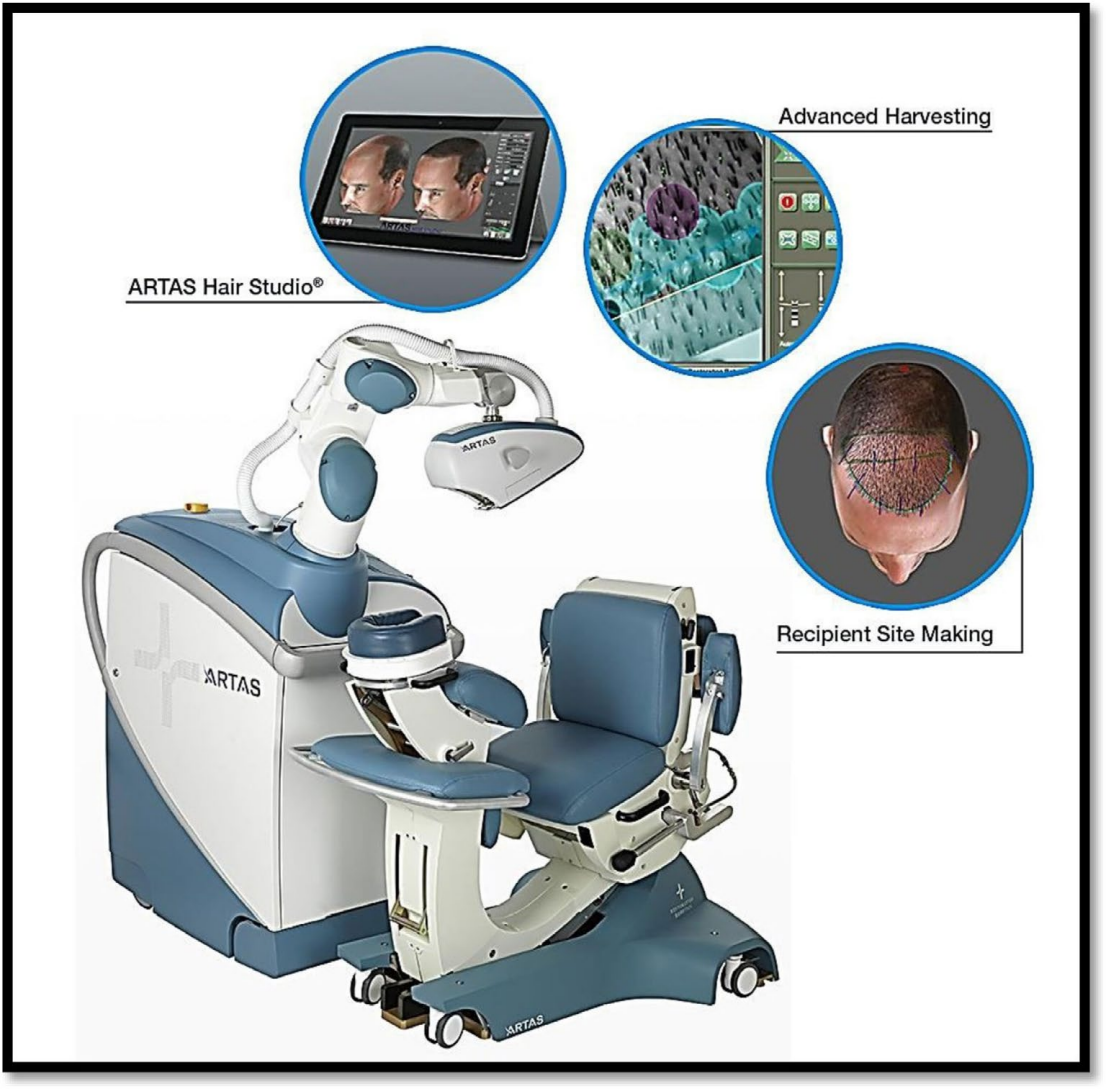
ARTAS robotic system for hair transplant [33].

AI‐based robotic systems are enhancing the precision and safety of aesthetic surgery, particularly for repetitive or detail‐oriented procedures [30]. In hair transplantation procedures, for instance, systems like the ARTAS Robotic System utilize computer vision to identify and harvest healthy follicular units with submillimeter accuracy, minimizing scarring and maximizing graft survival rates [31]. Similarly, AI‐guided lasers in skin resurfacing dynamically modulate energy levels in real time based on tissue response, optimizing efficacy while protecting surrounding areas [32]. These systems reduce human error, standardize outcomes, and allow surgeons to focus on advanced decision‐making, marrying robotic precision with human creativity to redefine procedural excellence (Figure 3) [34].

### 3.4 Patient Outcome Prediction

Predictive analytics based on AI are transforming risk analysis and outcome prediction in aesthetic medicine. By analyzing previous data from thousands of cases, algorithms predict how a patient will respond to procedures such as filler injections or liposuction based on variables like skin elasticity, healing capacity, and metabolic rates [35]. As an example, AI models may offer probability estimates of such complications as delayed swelling or asymmetry, enabling clinicians to adjust techniques or recommend alternative procedures. Such forward‐looking risk management not only enhances safety but also trust in patients through congruence of expectations with evidence‐based probabilities, encouraging informed consent and satisfaction over time [36].

### 3.5 Enhanced Patient Experience

AI is transforming the patient experience with intelligent tools that make interactions more efficient and insightful [37]. Chatbots and virtual assistants, like those embedded in clinic websites, offer 24/7 availability for booking consultations, addressing preprocedure questions, or monitoring recovery through symptom‐checking algorithms. Following procedures, AI‐powered apps deliver personalized aftercare reminders and monitor healing progress through photo submissions that are assessed using computer vision [38]. Education websites leverage generative AI to create patient‐friendly content, simplifying complex procedures like thread lifts or microneedling into simple terms through interactive videos or infographics. These features reduce administrative burdens on staff, empower patients with knowledge, and offer a seamless, easy experience from consultation through recovery (Table 1) [39].

**TABLE 1:** Key AI applications in aesthetic medicine.

Application	Example technologies	Benefits	Limitations
Facial analysis and simulation	ModiFace, Crisalix	Objective symmetry assessment; patient expectation management	Limited ethnic diversity in training data
Personalized treatment planning	Haut.AI, SkinMedica	Tailored regimens; reduced adverse effects	Requires extensive patient data input
Robotic‐assisted procedures	ARTAS Robotic System	Submillimeter precision; minimal scarring	High cost; limited procedural scope
Patient outcome prediction	AI‐driven risk assessment tools	Proactive complication mitigation	“Black box” decision‐making
Enhanced patient experience	AI chatbots, virtual assistants	24/7 support; streamlined workflows	Impersonal patient interactions

## 4 Challenges and Ethical Considerations

### 4.1 Data Privacy and Security

The use of AI in aesthetic medicine raises serious concerns about the privacy and security of sensitive patient information, particularly biometric data such as 3D facial scans, skin texture analysis, and genetic data. These data are highly personal and vulnerable to breaches, particularly when stored or processed by third‐party platforms. Unauthorized access can lead to identity theft, discrimination, or misuse of images for unethical purposes (e.g., deepfakes) [40]. Compliance with regulations like GDPR and HIPAA is critical, yet it is still possible to have loopholes in the acquisition of cloud‐based AI software and end‐to‐end encryption. Clinics must introduce robust cybersecurity measures and open data governance for maintaining patient confidentiality while harnessing the power of AI [41].

### 4.2 Bias and Algorithmic Fairness

Aesthetic medicine AI systems can perpetuate bias if they are trained on unrepresentative data. For instance, algorithms trained on predominantly lighter skin tones can misdiagnose conditions like hyperpigmentation in darker‐skinned patients, leading to suboptimal or even harmful recommendations [42]. Similarly, facial symmetry models would be founded on Western beauty standards, ignoring ethnic variation. Biases such as these devalue equity and potentially amplify disparities in the quality of care. Addressing this requires curation of diverse and inclusive training datasets, routine algorithmic review, and multidisciplinary participation in AI design, including ethicists and sociologists, to ensure that tools treat all patient groups equally [43].

### 4.3 Overreliance on Technology

Whereas accuracy is enhanced by AI, excessive dependence on algorithmic recommendations can work against clinical judgment. Aesthetic medicine is inherently one of personal choice—that is, balancing a patient's ideal with anatomical potential—calling for human intuition and empathy [44]. Excessive automation might lead to formulaic treatment with indifference to psychosocial or cultural concerns. For example, an AI could be set to prefer measurements of facial symmetry to a patient's personal idea of beauty [45]. Clinicians must learn to control, using AI as a tool to support decision‐making rather than a replacement for judgment, and acquire skills to critically analyze and interpret insights offered by AI [46].

### 4.4 Regulatory Hurdles

The swift development of AI in aesthetic medicine has been ahead of regulatory guidelines, leading to uncertainty regarding safety and efficacy standards. In contrast to drugs or medical devices, AI algorithms tend to develop dynamically, making it difficult for conventional approval procedures [47]. Regulatory agencies such as the FDA and EMA are establishing guidelines for AI/ML‐based software, but there are inconsistencies between regions [48]. For instance, a virtual simulation tool that has been approved in a country can be legally limited elsewhere due to differences in data privacy laws or clinical validation needs [49]. Global standardization and clear channels for tracking real‐world performance are required to ensure innovation while safeguarding patient health [50].

### 4.5 Patient Trust and Transparency

Building patient trust in AI‐supported aesthetic therapy relies on transparency about the contribution of algorithms to decision‐making. Patients are generally unaware that AI is being utilized in their therapy or may perceive it as impersonal or intrusive [51]. Clinicians must clearly define the purpose, limitations, and risks of AI algorithms—for example, that a virtual simulation constitutes a probability, not a guarantee. Informed consent processes should disclose data use and algorithmic involvement [52]. Furthermore, “black box” AI systems, which are unexplainable, can be barriers to trust. The use of interpretable models and educating the patient encourages collaboration, making the individual feel empowered rather than disconnected by technology in their aesthetic process (Table 2) [53].

**TABLE 2:** Ethical challenges and mitigation strategies.

Challenge	Risks	Mitigation strategies
Data privacy and security	Biometric data breaches; deepfake misuse	HIPAA/GDPR compliance; end‐to‐end encryption
Algorithmic bias	Underrepresentation of darker skin tones	Diverse training datasets; third‐party audits
Overreliance on AI	Erosion of clinical judgment	AI as decision‐support only; clinician training
Regulatory hurdles	Inconsistent global approvals	Harmonized FDA/EMA guidelines; real‐world monitoring
Patient trust issues	Lack of transparency in AI recommendations	Explainable AI (XAI); informed consent protocols

## 5 Future Directions

### 5.1 Integration With Emerging Technologies

The union of AI and augmented reality (AR), virtual reality (VR), and wearables will soon revolutionize patient experience and care delivery in aesthetic medicine [54]. AR/VR platforms, such as immersive consultation platforms, allow patients to “try on” the likely outcomes of procedures like facelifts or lip augmentation in real time, facilitating informed decision‐making [55]. Surgeons can utilize VR simulations to plan complex interventions, overlaying AI‐generated 3D mappings of anatomy onto a patient's physical features for precision [56]. Meanwhile, wearable technology with AI, be it smart patches or rings, monitors skin moisture, UV levels, or collagen levels in real time and enables anticipatory adjustments to skincare routines or treatments. These technologies integrate physical and virtual experiences, transforming consultations into in‐depth data, two‐way conversations, and allowing patients to actively participate in their aesthetic treatment [57].

### 5.2 Advancing Precision Medicine

AI is also releasing the promise of genomics and biomarkers to introduce precision into aesthetic treatments [58]. By analysis of genetic data linked to skin elasticity, wound healing, or susceptibility to pigmentation disorders, algorithms are able to predict individual responses to treatments like laser therapy or microneedling. AI models, for example, can identify patients who are likely to form keloid scarring, whereupon less invasive treatments would be indicated by clinicians [59]. Startups like SkinDNA and Nutrino employ AI to interpret genetic and metabolic profiles in order to design tailored regimens for anti‐aging or acne management [60]. This shift from generalized protocol to biology‐driven customization ensures interventions are aligned with a patient's unique physiological blueprint, maximizing efficacy and minimizing risks [61].

### 5.3 Ethical AI Development

As AI increasingly plays a central role in aesthetic medicine, ethical practices in development are necessary to reduce harm and achieve fairness. Building diverse datasets is done by intentional engagement with various groups to obtain representative skin tones, ethnic features, and age distributions—overcoming conventional biases of training data [62]. Transparent algorithms built with explainable AI (XAI) frameworks allow clinicians and patients to visualize how the recommendations are produced, increasing accountability [63]. Tools like IBM's AI Fairness 360 provide open‐source technology to audit and correct for bias. Ethicists, technologists, and clinicians must collaborate on creating standards that prioritize patient well‐being above business considerations and that result in AI tools maintaining dignity and cultural sensitivity as goals for pursuing beauty [50].

### 5.4 Global Collaboration

The complexity of AI integration necessitates cross‐border and cross‐sectoral cooperation to strike a balance between innovation and safety [64]. Players in the industry, such as Google Health and Perfect Corp, a startup, are collaborating with plastic surgeons and dermatologists to screen AI solutions for skin diagnosis or virtual modeling [65]. Concurrently, regulatory bodies such as the FDA and WHO are developing standardized testing protocols for AI‐enabled devices, balancing against rapid innovation and stringent regulation [66]. International networks, for example, the International Aesthetic Surgery Innovation Network, provide access to sharing information on best practices, stewardship of data, and ethical frameworks [67]. These networks ensure AI advancements to be scalable, culturally sensitive, and evidence‐driven, bridging the gap between innovative technology and real‐world use [68].

### 5.5 AI in Preventative Aesthetics

AI is shifting the focus of aesthetic medicine from repair to prevention by the identification of early signs of aging or skin damage. Predictive models analyze longitudinal data—lifestyle patterns, environmental exposures, and serial imaging—to forecast issues like collagen loss or hyperpigmentation prior to them being visibly apparent [69]. Apps like SkinVision use AI to assess moles for the risk of melanoma [70], while others like Olay's Skin Advisor examine selfies to recommend preventive skincare [71]. When these features are integrated with telehealth, clinicians are able to catch conditions earlier and offer individualized advice on sun avoidance, nutrition, or procedures that are minimally invasive. This forward‐looking strategy not only enhances long‐term outcomes but positions aesthetic medicine in concert with broader trends in preventive medicine, oriented toward wellness instead of overreactive intervention [72].

## 6 Results

The integration of AI into aesthetic medicine has led to transformative advancements across diagnostic, procedural, and patient care domains. Key findings from the literature include:

### 6.1 Enhanced Facial Analysis and Simulation

AI‐powered computer vision and deep learning can now perform high‐accuracy facial landmark detection, symmetry analysis, and aging prediction. Augmented reality (AR) and 3D modeling have also improved pretreatment planning by allowing patients to visualize realistic results, increasing satisfaction and decision making.

### 6.2 Personalized Treatment Planning

Machine learning algorithms deliver hyper‐personalized recommendations according to the genetic data, skin types, and medical history. Haut.AI and SkinMedica are tools that optimize efficacy and safety through minimizing trial‐and‐error and matching treatments to individualized biological profiles.

### 6.3 Robotic‐Assisted Procedures

AI‐powered robotic systems like ARTAS have improved accuracy and reproducibility in aesthetic procedures like laser and hair transplantation. AI‐driven real‐time feedback minimizes room for error, optimizes outcomes, and enhances the safety of procedures.

### 6.4 Predictive Outcome Modeling

Predictive analytics allow clinicians to foresee treatment outcomes and complication risks, refining risk stratification and aligning patient expectations with likely outcomes. They facilitate informed consent and active clinical decision‐making.

### 6.5 Improved Patient Experience

AI‐powered virtual assistants, chatbots, and learning platforms have optimized administrative workflows and improved patient engagement. Monitoring of procedure recovery and customized aftercare through AI apps has minimized clinical workload and improved patient satisfaction.

### 6.6 Ongoing Challenges

Although the area evolves, the practice faces ethical as well as practical problems, including: (1) Security and privacy issues in the handling of biometric data. (2) Biased computation algorithms for the analysis of non‐Western face phenotypes or darker‐skinned patients. (3) Reliance on AI to the point where it detracts from human common sense and medical judgment. (4) Regulative uncertainty causing universal rollout extension. (5) Issues concerning trustworthiness and transparency, especially with regard to “black box” designs.

### 6.7 Future Directions

1. Integration with AR/VR and wearables to deliver immersive consultation and real‐time monitoring. 2 Spreading precision medicine through genomic and biomarker‐based AI models. 3. Ethical AI development, promising fairness, inclusivity, and interpretability. 4. International cooperation between tech companies, clinicians, and regulators to synchronize standards. 5. Preventive aesthetics through predictive AI that can predict and proactively delay signs of aging.

## 7 Discussion

AI use in aesthetic medicine is a paradigm shift in clinicians' assessment, planning, and delivery of treatments [22]. The article highlights the revolutionary impact of AI in many aspects of practice, ranging from facial analysis and customized planning to robot‐assisted treatment and predictive modeling. The benefits are vast but accompanied by an array of challenges that need to be considered seriously to enable ethical and effective integration [23, 24].

One of the most powerful strengths of AI is its ability to enhance diagnostic precision and objectivity [32]. Deep learning and computer vision technologies now outperform traditional visual inspection in the identification of facial asymmetries, skin conditions, and aging signs. This has resulted in more precise and reproducible treatment strategies. In addition, the introduction of 3D simulations and augmented reality has improved patient‐practitioner communication, balancing aesthetic expectations with realistic outcomes [27].

These technologies do more than improve clinical judgment; they extend the limits of what may be perceived, creating a better and wiser consultation process [38].

Another urgent innovation is hyperpersonalized treatment planning. Computer algorithms, through analyzing massive pools of information—genetics, lifestyle, and history of treatments—enable clinicians to shift beyond cookie‐cutter protocols to biological‐based treatments [28]. This reduces side effects, amplifies efficacy, and facilitates patient‐centered care. Still, the quality and representativeness of training data remain essential. If datasets are not representative of different ethnicities, ages, and skin colors, the produced AI products will unintentionally reinforce biases and create disparities in care quality [29].

The application of AI‐powered robotic systems has also improved procedure consistency, particularly in long or precision‐based procedures like hair transplantation and laser resurfacing [32]. Not only do these systems deliver maximum technical performance, but they also allow clinicians to spend more time on higher level decision‐making and artistry. However, the arrival of such automations brings with it fear of reliance on technology, which could undermine the clinician's input to subjective aesthetic judgment—a domain where human experience and empathy cannot be replaced by anything else [34].

Predictive analytics provide a further level of advantage, enabling practitioners to foresee likely treatment outcomes and potential complications. This application enhances risk stratification and promotes transparency in patient counseling [35]. However, many AI devices remain “black boxes,” providing answers but with minimal knowledge of how the decision was reached. This absence of transparency has the potential to undermine patient trust and clinical accountability, especially in elective procedures where expectations are by nature subjective and emotionally laden [36].

In general, the regulatory and ethical landscape for AI in aesthetic medicine remains under development [46]. Cybersecurity and data protection remain at the forefront, particularly with the sensitive nature of facial images and biometric data. Regulatory frameworks, such as the FDA's evolving SaMD guidelines for software as a medical device, are beginning to address these shortcomings, but as yet, no global standardization exists. With continued advancements in AI technologies, there is an ever‐growing need to embrace harmonized worldwide regulation, open validation protocols, and performance monitoring in the field [45].

Also, transparency and patient education are essential to the building of trust in AI‐based care. Clinicians must explain how AI systems aid in treatment planning, as well as their limitations and probabilistic nature [50]. Combining XAI systems and ethical regulation can close the gap between high‐tech capability and patient understanding, facilitating informed consent and shared decision‐making [63].

In the years to come, the convergence of AI with technologies such as wearable sensors, genomics, and virtual/augmented reality could further expand the realm of aesthetic medicine [70]. These developments enable preventative approaches, which allow early detection and intervention before clinical evidence of aging is evident. For this to be optimized, however, ongoing multidisciplinary effort among clinicians, engineers, ethicists, and regulators has to be continued [72].

Briefly put, AI will enhance aesthetic medicine through greater accuracy, individualization, and prognostic treatment. Its introduction must be founded on ethical considerations, universal design, and continued clinical observation. Discovering an optimal synthesis of technological innovation and the irreplaceable human aspects of aesthetic practice will be crucial to making AI an enabler rather than a displacer.

## 8 Conclusion

AI is unequivocally transforming aesthetic medicine, delivering innovative facial analysis tools, personalized treatment planning, robotic precision, and predictive patient care. From AI‐powered simulations that enhance consultations to genomics‐driven skincare regimens, these innovations are reshaping beauty, safety, and efficacy standards. As the specialty further evolves, however, concerns regarding data privacy, algorithmic bias, and regulatory gaps must be proactively addressed to ensure ethical and equitable use. We must have a shared call to action—clinicians, technologists, regulators, and ethicists must collaborate together to promote responsible innovation. Investment in diverse AI development, robust regulatory frameworks, and patient education will be instrumental in guiding this technological revolution. Clinics and developers must invest in transparency so that AI augments—rather than replaces—the human touch that remains at the center of aesthetic care. Ultimately, the future of aesthetic medicine is about achieving a balance—harnessing the analytical capability of AI without sacrificing the artistry, empathy, and customized judgment that characterize the specialty. By synthesizing innovative technology with human‐focused values, the field can create unparalleled opportunities, with advancements working not only toward aesthetic ideals but overall patient health. The path forward is as much about advancement as it is about integrity, sculpting a future where beauty and technology unfold together.

## Author Contributions

M.S.A.D., G.F.M., L.M.A., S.S.B., A.M.A.D., and A.M.B. collected the materials. M.S.A.D., G.F.M., L.M.A., S.S.B., A.M.A.D., and A.M.B. did the analysis and wrote the paper. M.S.A.D., G.F.M., L.M.A., S.S.B., A.M.A.D., and A.M.B. wrote the manuscript. M.S.A.D., G.F.M., L.M.A., S.S.B., A.M.A.D., and A.M.B. did the tables.

## Conflicts of Interest

The authors declare no conflicts of interest.

## Data Availability

The data that support the findings of this study are available from the corresponding author upon reasonable request.
